# Expressed emotions and caregiving appraisals among relatives of patients with psychotic disorders: a cross-sectional study

**DOI:** 10.1186/s12888-026-07868-7

**Published:** 2026-02-04

**Authors:** Irene Norheim, Jan Ivar Røssberg, Maria Lie Selle, Kristen Woodberry, Mary O’Brien, Reidar Pedersen, Lars Hestmark, Roar Fosse, Kristiane Myckland Hansson, Kristin Heiervang, Maria Romøren

**Affiliations:** 1 Division of Mental Health and Addiction, Department of Mental Health Research and Development, Vestre Viken Hospital Trust, Drammen, Norway; 2 Division of Mental Health and Addiction, Section for Treatment Research, Oslo University Hospital, Oslo, Norway; 3 Institute of Clinical Medicine, University of Oslo, Oslo, Norway; 4 Health Services Research Unit, Akershus University Hospital, Lørenskog, Norway; 5 Center for Clinical and Translational Science, Maine Health Institute for Research, Scarborough, Maine USA; 6 Tufts University School of Medicine, Boston, Massachusetts USA; 7 Department of Psychology, Yale University, New Haven, Connecticut USA; 8 Institute of Health and Society, Centre for Medical Ethics, University of Oslo, Oslo, Norway; 9 Division of Mental Health Services, Akershus University Hospital, Lørenskog, Norway

**Keywords:** Relatives, Caregiving appraisals, Expressed emotion, Quality of life, Health support, Psychosis, Schizophrenia

## Abstract

**Background:**

Relatives’ expressed emotions (EE) and caregiving appraisals are associated with the prognosis of individuals with psychotic disorders as well as relatives’ own well-being. The main aim of the present study was to examine whether sociodemographic factors, patients’ clinical characteristics, relatives’ health and quality of life (QoL), and relatives’ perceived support explained a significant amount of the variance in expressed emotion (EE) and caregiving appraisals among relatives of patients with psychotic disorders.

**Methods:**

Baseline data from *The Implementation of guidelines on Family Involvement for persons with Psychotic disorders* (IFIP) trial were compiled from 231 patient-relative pairs across 15 Community Mental Health Centres (CMHC). Relatives completed assessments on EE; emotional overinvolvement (EOI) and critical comments (CC) (Family Questionnaire), negative and positive caregiving appraisals (Experience of Caregiving Inventory), health and QoL (Care Related Quality of Life), healthcare professional support (Caregiver Well-being and Support) and sociodemographic factors. Patients reported on their own difficulties with mental health and functioning (Behavior and Symptom Identification scale) while clinicians assessed patients’ functioning (Global Assessment of Functioning) and sociodemographic factors. Pearson and Spearman correlations and hierarchical multiple linear regressions were used for statistical analyses.

**Results:**

Duration since first psychosis diagnosis, patients’ mental health and functioning, and relatives’ health problems explained a substantial proportion of variance in relatives’ EOI and negative caregiving appraisals. Together with household income level, duration since first psychosis diagnosis explained a significant amount of the variance in positive caregiving appraisals. Relatives’ perceived support from healthcare professionals explained a significant amount of the variance in CC and negative caregiving appraisals, even after adjusting for contextual variables.

**Conclusion:**

Psychotic disorders entail significant burdens and shape the family climate for both patients and their relatives. Negative caregiving appraisals and EOI may reflect normal reactions to the responsibilities and challenges relatives face. EOI and CC, however, may be associated with distinct factors, necessitating tailored psychoeducational and support interventions. The findings suggest that relatives’ perceived support from healthcare professionals could have a substantial positive impact on CC and negative caregiving appraisals, which is important for clinicians to recognise and address.

## Introduction

Relatives play a vital role in the early detection of illness and establishment of contact with healthcare services for individuals in the early phase of psychotic disorders [1, 2]. The substantial reduction in specialized inpatient mental health services and the shift to community care in recent decades have necessitated family involvement in treatment, rehabilitation and caregiving [3–6]. Extensive research demonstrates that systematic family interventions (FIs) may reduce symptoms and inpatient stays [7–10], lower relapse rates [11–14], and improve social and global functioning [8, 9, 11, 12, 15, 16], as well as quality of life [8, 17]. In addition, FIs may decrease levels of expressed emotions (EE) and burden among family members [11, 12, 18–21]. FIs may include psychoeducation, crisis intervention, emotional support and training in coping skills [22], delivered through family psychoeducation (FPE) programmes [23], for example psychoeducational multi-family groups (PMFG) [24], behavioural family therapy (BFT) [25, 26] and family‑focused therapy (FFT) [27]. The Norwegian national treatment guidelines for psychotic disorders recommend FPE in multi-family or single-family groups as a first‑line treatment [28] (currently under review). The national guide for relatives in health and care services [29] likewise recommends basic family involvement and support (BFIS) for all relatives, irrespective of the patient’s or service user’s diagnosis.

Caregiver burden for relatives of persons with psychotic disorders appear to be higher when compared with caregivers of persons with other mental illnesses and conditions [30–32], leading to elevated distress, anxiety and depression [5, 33–36], while also increasing the risk of PTSD and burnout [37, 38]. This may result in a reduction in caregiving, a deterioration in quality of life, and a decline in both physical and mental health [31, 39–44], thereby necessitating recovery-oriented services for relatives as well [45].

Despite the emphasis on the negative aspects of caregiving for individuals with psychotic disorders, studies indicate that relatives also experience significant positive gains. These may include becoming more sensitive to persons with disabilities, family solidarity, achieving greater clarity about their priorities in life, and developing a stronger sense of inner strength and personal growth [46, 47]. Formal support from mental health professionals, through information sharing and collaborative practices, as well as informal support from family members and peer support groups, are significantly associated with more positive caregiving experiences [48].

To encompass the entire caregiving experience, not just the ‘burden’ [5], Szmukler et al. conceptualized caregiving based on Lazarus and Folkman’s ‘stress-appraisal-coping’ paradigm [49]. They developed a self-report measure, The Experience of Caregiving Inventory (ECI), assessing caregivers’ negative as well as positive appraisals [50]. Caregiving appraisal is the process by which a caregiver evaluates the significance of caregiving, taking into account the nature of the stressor as well as their available coping resources [46]. Negative caregiving appraisals are also referred to as burden of care [51]. According to the stress-appraisal-coping model, patient symptoms and disability increase negative caregiving appraisal, while social support and service inputs reduce them [52].

One of the most influential concepts in psychosocial research on psychosis has been EE. The way caregivers understand and react when their loved one develops psychosis influences both the course of the illness [53–58] and the caregivers’ own well-being [59, 60]. EE assesses the following domains of interpersonal relationships: critical comments (CC)/hostility, and emotional overinvolvement (EOI) [61]. High levels of EE are regarded as a form of psychosocial stress for the patient [62], and the relatives’ level of EE is influenced by a variety of factors, such as specific personality traits (for example, inflexibility), the way they perceive and attribute patient behaviour and symptoms, and the patient’s vulnerability to stress [53, 62–64]. The EE concept has been criticised for holding families responsible for the course of a relative’s mental disorder [65, 66]. A more balanced view is that EE behaviors, such as criticism and intrusiveness, may stem from caring for a severely mentally ill person without adequate knowledge, guidance or support [26, 67, 68]. Finally, it is important to emphasize that many families of individuals with psychotic disorders do not exhibit high levels of EE and in fact demonstrate warmth and provide positive remarks and encouragement to their ill relative.

Nonetheless, given the significance of EE and caregiving appraisals for the trajectory of patients’ illness and relatives’ well-being, it is essential to gain a deeper understanding of how various aspects - such as sociodemographic factors, clinical characteristics, relatives’ health and QoL, and caregiver support systems - contribute to both the positive and negative dimensions of family climate and care. Measuring these aspects through self-report can facilitate the tailoring of therapeutic interventions to the needs of individuals with psychotic disorders and their families. To our knowledge this is the first study performing multiple analyses on both EE and caregiving appraisals simultaneously, specifically examining the importance of professional support in influencing these family responses [9, 17, 19].

More specifically we wanted to investigate:

1. How do patients’ sociodemographic factors and clinical characteristics correlate with their relatives’ EOI, CC, and negative and positive caregiving appraisals?
2. How do relatives’ sociodemographic factors, health and QoL, and perceived support correlate with EOI, CC, and negative and positive caregiving appraisals?
3. To what extent do sociodemographic factors, patients’ clinical characteristics, and relatives’ health and QoL explain variance in EOI, CC, and negative and positive caregiving appraisals, and to what extent do relatives’ perceived support add further explanation after controlling for these contextual variables?

## Methods

### Study design

This study adopted a descriptive, cross-sectional design using baseline data collected from the *Implementation of guidelines on Family Involvement for persons with Psychotic disorders* (IFIP) trial [69]. The IFIP trial was a cluster randomized controlled trial where 15 community mental health centre (CMHC) units in South-Eastern Norway were randomly allocated to either an Implementation Support Program (ISP; experimental arm) or treatment as usual (control arm).

### Participants and procedure

The participating CMHC units were asked to include patients with a psychotic disorder together with one of their closest relatives. For patients, the inclusion criteria were: an established or tentative psychotic disorder diagnosis (F20–29 in ICD-10) certain enough to begin treatment; age *≥* 18 years. The diagnoses were made by clinicians at the CMHCs. The exclusion criteria were: being a forensic client; not being competent to consent to participation; not having any relatives; having previously completed more than five sessions of family psychoeducation (FPE) in single-family treatment or more than ten sessions of FPE in multi-family groups or a similarly structured FI. For relatives, the inclusion criteria were: being a relative of a patient with a diagnosis as described above; age *≥* 18 years.

The recruitment period was from March 2019 to September 2020. Patients who agreed to participate in the IFIP trial provided written informed consent to participate and for the clinician to contact a close relative. Subsequently, relatives were contacted and interested relatives provided written informed consent. At the outset of the IFIP trial, each CMHC unit had between 28 and 217 patients with psychotic disorders, with 1392 patients combined. In total, 231 patient/relative pairs were recruited to this study.

Parts of the recruitment period coincided with the COVID‑19 pandemic, and 12 (5%) of the 231 pairs were recruited after the outbreak on 12 March 2020. The EE and ECI scores for these 12 relatives did not differ from those of relatives recruited before the outbreak, and they were therefore included in the analyses of the total data material.

### Instruments

#### Basic information

Relatives reported on sociodemographic factors such as age, gender (male; female), civil status (married/partner; single/divorced/widowed), education level (secondary school or less; higher education), working status (unemployed or retired; employed (full or part time); and household annual income. The household annual income in Norwegian kroner was used as an ordinal variable based on 9 level categorial variables from less than 200 000 to more than 900 000. They also reported on their relationship with the patient (parent; married/partner; other (including child or sibling)), and whether the relative lived with the patient.

Clinicians reported the patients’ sociodemographic factors such as age, gender (male; female), civil status (married/partner; single/divorced/widowed), living arrangement (alone, other, with partner), and employment (unemployed; employed (full or part time); other). They also reported the number of years since the patient was diagnosed with a psychotic disorder (F20-29, ICD-10).

#### Family questionnaire (FQ)

The FQ [70] was used to measure relatives’ expressed emotion (EE). This is a 20-item self-report measure with two subscales, emotional overinvolvement (EOI) and critical comments (CC), with 10-items in each subscale. Each item is measured on a 4-point scale (1 = never/very rarely to 4 = very often). Higher scores indicate higher levels of EE. The cut-off of EOI is low < 27, and high ≥ 27. The cut-off of CC is low < 23, and high ≥ 23. The FQ has shown a distinct factor structure, strong internal consistency within its subscales, and good concurrent validity when compared to the widely used Camberwell Family Interview [71]. In the current study, the Cronbach’s α was 0.83 EOI and 0.88 for CC.

#### Experience of caregiving inventory questionnaire (ECI)

Negative and positive caregiving appraisals were measured using the ECI [50, 52]. It is a self-report questionnaire that assesses caregivers’ appraisals of the burden associated with their role over the past month. The ECI comprises 66 items and 10 subscales, distributed on a negative personal experience scale: difficult behaviors, negative symptoms, stigma, problems with services, effects on the family, loss, dependency, and need to back-up, as well as a positive personal experience scale: positive personal experience, and good aspects of the relationship with patient. Each item is rated on a 5-point scale (0 = never to 4 = nearly always) where a higher score on the negative scale reflecting more negative appraisals, and a higher score on the positive scale reflects more positive appraisals. In the current study, Cronbach’s α for the ECI ranged from 0.70 (good aspects of relationships) to 0.88 (negative symptoms).

#### The care related quality of life questionnaire (CarerQoL-7D)

The CarerQoL-7D [72] was used to measure the current impact of caregiving on relatives’ health and QoL at the moment. It is a self-report questionnaire with 7-items that describe the caregiving situation, each scored as “no”, “some”, or “a lot” (CarerQol-7D). One of the descriptions is as follows: I have no/some/a lot of problems with my own mental health (e.g., stress, anxiety, low mood, depression, concern about the future).

In addition, the form includes a validating question regarding general well-being with scores from zero (completely unhappy) to ten (completely happy) (CarerQol-VAS). The CarerQol was developed for efficient evaluation of healthcare interventions affecting caregivers and has also demonstrated positive results in tests for clinical validity [72–74]. In the current study, the Cronbach’s α for the CarerQoL was 0.73.

#### Carer Well-being and support questionnaire version 2, part B - support scale (CWS-B)

Relatives’ perceived support from healthcare professionals was measured using the CWS-B [75]. It is comprised of 17 items and 3 subscales: information and advice for carers, their involvement in treatment and care planning, and support from medical and/or care staff. Relatives rated each item on a 4-point scale with scores from zero “very dissatisfied” to three “very satisfied”. The CWS-B has shown moderate acceptability and good reliability [75, 76]. Validity testing for the support scale is limited by the lack of appropriate validating measures. In the current study, the Cronbach’s α for the “information and advice for carers” was 0.92, for “your involvement in treatment and care planning” it was 0.94, and for “support from medical and/or care staff” it was 0.94.

#### Global assessment of functioning (GAF) – Split version

Patients’ overall symptoms and functioning were measured with the 2 items clinician-rated GAF – Split version, which separates symptoms and functioning into two ratings [77]. The scores ranging from one to one hundred, where lower values mean more severe symptoms/functional impairments. The GAF has demonstrated varying interrater reliability, but it has been shown to be acceptable when raters are experienced and trained [78, 79]. The validity has been found to be modest to good [80, 81].

#### Behavior and symptom identification scale (BASIS-24)

The BASIS-24 was used to measure patients’ general experience of mental health and functioning. The BASIS-24 [82] is a 24-item self-report measure with six sub-scales: depression/functioning, emotional lability, self-harm, difficulty in interpersonal relationships, substance abuse, and psychosis. Items are rated on a scale from zero (not difficult/none of the time/never) to four (very difficult/all the time/always). The BASIS-24 scale has demonstrated good construct validity and internal consistency [82–84]. In the current study, Cronbach’s α for the BASIS-24 ranged from 0.70 (emotional lability) to 0.89 (depression/functioning).

### Statistical analyses

Analyses were conducted using R version 4.2.3. Pearson correlations (or Spearman if appropriate) were used to examine the associations between each continuous independent variable and outcome variables EE (EOI and CC) and ECI (negative scale and positive scale). For categorical independent variables, t-test or one-way ANOVA was used to test the outcome variable means. From t-tests we present the test statistic and its p-value, and from ANOVA we present the proportion of variance explained (η²) and the corresponding p-value.

To explore whether support for families helps to explain critical aspects of family environment, after controlling for sociodemographic factors and clinical characteristics, we conducted a hierarchical regression analysis with perceived formal support as the last stage.

The hierarchical regression was used to estimate associations between the dependent and pre-selected independent variables in seven stages, and to examine additional variance explained by each stage. The seven stages and their variables were based on the research questions and the bivariate associations. To reduce multicollinearity, variables with strong intercorrelations (Pearson’s *r* > 0.7), were avoided.

Stage 1 contained years since diagnosis and patient gender, and stage 2 contained the relative variables age, gender, household income and education. Stage 3 contained GAF, stage 4 contained BASIS-24 sum, stage 5 contained CarerQoL: relatives’ problems with physical (none, some, many) and mental health (none, some, many). Stage 6 contained CarerQoL: relatives’ perceived support from family, friends, etc. (none, some, much), and stage 7 contained relatives’ perceived support from healthcare professionals (CWS-B).

We presented the results as estimated regression coefficients, standard deviations and p-values for stage 7, and the change in adjusted coefficient of determination ($\:{R}_{adj}^{2}$) for each stage. Residual plots were evaluated graphically to assess model assumptions, which were found to be fulfilled, and variance inflation factor (VIF) was computed for each independent variable value to detect multicollinearity, which was not present in the models. All tests were two-sided, with 0.05 significance level. As the goal was to identify potentially interesting relationships and pattens (that may inform future research), rather than to confirm predefined hypotheses, a p-value < 0.05 was considered indicative of potentially meaningful associations or trends.

## Results

### Descriptive analysis

A total of 231 patient-relative pairs were included. Table 1 presents the sociodemographic factors, relative-related characteristics, and the years since the patients were first diagnosed with a psychosis. Two-thirds of the relatives were parents to the patient, 14% were married to or in a partnership with the patient, and 20% were other relatives, such as a child or sibling. One-third of the relatives lived with the patient. The average age of the relatives included in the study was 54.6 years, with a majority being female (67%), and either married or living with a partner (70%). Three-fifths of the relatives were employed full-time or part-time. The average age of the patients was 36 years, with 58% being male. Over half of the patients lived alone, 17% lived with a partner, and 28% lived with others. The percentage of patients who were employed either full-time or part-time was 22%. Some of these received financial support from the Norwegian Labour and Welfare Administration. The mean duration since patients were first diagnosed with a psychotic disorder was 8.9 years, ranging from 3 months to 54 years.

**Table 1:** Description of sociodemographic factors, relative-related characteristics, duration since the patients’ first psychosis diagnosis and the different diagnosis

*N* = 231	*N* (%), mean (SD), median (IQR)
**Relative sociodemographic factors**	
Age, (1 missing), mean (SD)	54.6 (13.4)
Gender, N (%),	
Male	77 (33.3)
Female	154 (66.7)
Civil status (3 missing), N (%)	
Married/partner	160 (70.2)
Single/divorced/widowed	68 (29.8)
Educational level (3 missing), N (%)	
Secondary school or less	100 (43.1)
Higher education	128 (56.3)
Working status (4 missing), N (%)	
Unemployed or retired	85 (37.9)
Employed (full or part time)	142 (62.1)
Household annual income level in *NOK* (9 missing), median (IQR)*	600k-700k (500k)
**Relative-related characteristics**	
Relation to the patient (2 missing), N (%)	
Parent	151 (65.9)
Married/partner	33 (14.4)
Other (incl. child, sibling)	45 (19.7)
Living with the patient (2 missing), N (%)	
Yes	74 (32.3)
No	155 (67.7)
**Patient sociodemographic factors**	
Age, mean (SD)	36 (12.55)
Gender, N (%)	
Male	134 (58)
Female	97 (42)
Living arrangement (3 missing), N (%)	
Alone	126 (55.3)
Other	63 (27.6)
With partner	39 (17.1)
Main activity the last 6 month, N (%)	
Unemployed	123 (53.2)
Employed (full or part time)**	50 (21.6)
Other	58 (25.1)
Income from the Norwegian Labour and Welfare Administration, N (%)	122 (52.8)
**Duration since first psychosis diagnosis**	
Years since diagnosed, mean/SD	8.9 (9.3)
**Diagnosis**,** N (%)**	
Core schizophrenia (F20,21,22,25)	192 (83.1)
Acute and transient psychosis (F23)	18 (7.8)
Other psychosis (F28, 29)	21 (9.1)

N: number. SD: Standard deviation. IQR: Interquartile range. * NOK = Norwegian kroner. Household annual income groups; 1: 200 or less, 2: 200–300, …8: 800–900, 9: more than 900. ** *Employed (full or part time)* includes full-time, part-time, or qualification program under the auspices of the Norwegian Labour and Welfare Administration

Table 2 presents descriptive statistics for relatives’ EE, caregiving appraisals, health and QoL, healthcare professional support, and patients’ mental health and functioning. For the ECI negative and positive scale, the scores were relatively symmetric around the mean scores, ranging between 2 and 180 for the negative scale, and 2 and 51 for the positive scale. The EE scores were generally low, as only 29/228 (12.7%) of the relatives scored above the threshold for overinvolvement (scoring ≥ 27 on FQ, EOI subscale), and 31/226 (13.7%) of the relatives scored above the threshold for criticism (scoring ≥ 23 on FQ, CC subscale). The proportion of relatives which scored above the threshold for both overinvolvement and criticism was 11/226 (4.9%).

**Table 2:** Descriptive statistics for relatives and patients

Variable	Scale	*N*	Mean (SD)	Range
**Relative measurements**				
Caregiving appraisals^a^				
Positive caregiving appraisals	ECI Positive scale	230	26.52 (7.9)	0–56ꜛ
Negative caregiving appraisals	ECI Negative scale	230	70.67 (31.97)	0-208ꜜ
Expressed emotion^b^				
Emotional overinvolvement (EOI)	Family Questionnaire EOI	228	21.47 (4.69)	10–40ꜜ
Critical comments (CC)	Family Questionnaire CC	226	17.08 (5.28)	10–40ꜜ
Quality of life^c^	CarerQoL-VAS	230	6.57 (1.83)	0–10ꜛ
	1) CarerQoL	226	1.44 (0.59)	0–2ꜛ
	2) CarerQoL	229	1.52 (0.54)	0–2ꜛ
	3) CarerQoL	230	1.48 (0.57)	0–2ꜛ
	4) CarerQoL	229	1.59 (0.58)	0–2ꜛ
	5) CarerQoL	230	1.76 (0.48)	0–2ꜛ
	6) CarerQoL	228	0.91 (0.71)	0–2ꜛ
	7) CarerQoL	230	1.4 (0.64)	0–2ꜛ
Healthcare professional support^d^	CWS Support scale	222	31.56 (13.17)	0–51ꜛ
**Patient measurements**				
Mental health and functioning^e^	BASIS-24 overall mean score	228	1.17 (0.7)	0–4ꜜ
Functioning^f^	GAF-F	213	51.5 (12.6)	0-100ꜛ
	GAF-S	214	52.4 (11.9)	0-100ꜛ

Presented descriptive statistics are Mean (SD). Range: ꜜ= Higher value, negative outcome, ꜛ=Higher value, positive outcome

^a^The Experience of Caregiving inventory questionnaire (ECI). ^b^The Family questionnaire (FQ). ^c^The Care Related Quality of Life questionnaire (Carer QoL). Carer QoL-VAS (Visual Analogue Scale), and each question: (1) Fulfilment with carrying out my care tasks, (2) Relational problems with the care receiver, (3) Problems with my own mental health (e.g.,* stress*,* fear*,* gloominess*,* depression*,* concern about the future*), (4) Problems combining my care tasks with my daily activities, (5) Financial problems because of my care tasks, (6) Support with carrying out my care tasks, when I need it, (e.g. from family, friends, neighbors, acquaintances), (7) Problems with my own physical health (e.g.,* more often sick*,* tiredness*,* physical stress*)

^d^Carer Well-being and Support questionnaire version 2, part B - Support scale (CWS-B). ^e^The Behavior and Symptom Identification scale (Basis-24). ^f^The Global assessment of functioning scale (GAF), split versions for symptoms (GAF-S) and functioning (GAF-F)

### Correlational analysis

Correlations between EE and caregiving appraisals were as follows: The ECI negative scale was significantly related to EOI (*r* = 0.79, *P* < 0.001) and CC (*r* = 0.64, *P* < 0.001); The ECI positive scale was significantly related to EOI (*r* = 0.32, *P* < 0.001) but not to the CC (*r* = -0.02, *p* = 0.79); EOI and CC were moderately correlated (0.51, *P* < 0.001); The ECI positive and negative scales were low/moderately correlated (*r* = 0.27, *P* < 0.001).

Table 3 (patient factors) and Table 4 (relative factors) presents correlations for continuous variables and t-test or one-way ANOVA-tests for categorical variables.

**Table 3:** Correlations of patient demographics and patients’ mental health and functioning with EE and caregiving appraisals

	Family questionnaire (FQ)					Experience of caregiving (ECI)				
		EOI		CC			Negative scale		Positive scale	
	N	Mean (SD)	r/T/η²	Mean (SD)	r/T/η²	N	Mean (SD)	r/T/η²	Mean (SD)	r/T/η²
Age	228		-0.11		0.08	230		-0.13*		-0.15*
Gender			1.94		0.51			2.51*		1.76
Male	132	21.98 (4.69)		17.24 (5.37)		134	75.09 (31.89)		27.3 (7.76)	
Female	96	20.77 (4.63)		16.88 (5.16)		96	64.51 (31.22)		25.44 (8.01)	
Living arrangements			0.01		0.01			0.01		0.03*
Other	125	21.98 (3.95)		16.19 (5.21)		126	72.81 (31.62)		28.94 (7.46)	
Alone	63	21.30 (4.84)		17.38 (5.26)		63	71.79 (31.97)		25.86 (7.4)	
With partner	37	21.11 (5.03)		17.61 (5.51)		38	62.47 (30.33)		25.66 (8.88)	
Main activity			0.05**		0.02			0.06**		0.01
Unemployed	122	22.39 (4.37)		17.84 (5.15)		123	77.62 (30.62)		27.1 (7.39)	
Employed	49	20.14 (5.12)		16.06 (4.80)		49	61.2 (29.76)		25.27 (7.68)	
Other	57	20.67 (4.63)		16.33 (5.75)		58	63.95 (33.57)		26.36 (9.06)	
Years since diagnosed	228		-0.14*		0.05	230		-0.13*		-0.18**
BASIS-24^a^	225		0.21**		0.14*	227		0.21***		0.05
GAF-F^b^	210		-0.23***		-0.18**	212		-0.31***		-0.02
GAF-S^b^	210		-0.18**		-0.18**	212		-0.26***		0.07

*P ≤ 0.05; **P ≤ 0.01; ***P ≤ 0.001. SD: standard deviation. r: correlation coefficients. T: T-statistics (for variables with two categories). η²: one-way ANOVA effect size (for variables with three categories). EOI: Emotional overinvolvement, CC: Critical comments. ^a^The Behavior and Symptom Identification scale (Basis-24). Overall mean score. ^b^The Global assessment of functioning scale (GAF), split versions for symptoms (GAF-S) and functioning (GAF-F)

**Table 4:** Correlations of relative demographics, relatives’ health and QoL, and relatives’ perceived support with EE and caregiving appraisals

	Family questionnaire (FQ)					Experience of caregiving (ECI)				
		EOI		CC			Negative scale		Positive scale	
	N	Mean (SD)	r/T/η²	Mean (SD)	r/T/η²	N	Mean (SD)	r/T/η²	Mean (SD)	r/T/η²
Age	228		-0.03		-0.09	230		-0.03		-0.02
Gender			3.9***		0.27*			2.45*		3.41**
Female	152	22.32 (4.44)		17.15 (5.17)		154	74.38 (30.52)		27.85 (6.92)	
Male	76	19.78 (4.74)		16.95 (5.50)		76	63.17 (33.7)		23.83 (9.05)	
Civil status			1.19		-1.08			1.17		1.57
Married/partner	159	21.23 (4.78)		17.34 (5.54)		160	69.21 (31.96)		26 (7.75)	
Single/divorced/ widowed	67	22.03 (4.53)		16.56 (4.64)		68	74.65 (32.10)		27.84 (8.25)	
Educational level			2.22*		2.11*			2.67**		1.31
Secondary school or less	98	22.27 (4.88)		17.96 (5.53)		100	77.11 (30.66)		27.32 (7.70)	
Higher education	128	20.86 (4. 51)		16.45 (5.02)		128	65.92 (32.34)		25.95 (8.08)	
Working status			1.19		-0.35			1.18		0.92
Unemployed or retired	84	21.92 (4.79)		16.89 (5.21)		85	73.65 (33.38)		27.18 (7.72)	
Employed	141	21.14 (4.65)		17.15 (5.34)		142	68.41 (30.64)		26.19 (8.06)	
Household income	220		-0.18**		0.01	222		-0.17*		-0.19**
Relation to patient			0.01		0.01			0.02		0.01
Parent	150	21.83 (4.54)		17.36 (4.95)		151	74.01 (31.25)		27.09 (7.47)	
Married/partner	32	20.72 (5.15)		18.11 (5.62)		33	62.15 (30.83)		25.88 (9.43)	
Other	45	20.80 (4.88)		16.73 (5.24)		45	66.49 (34.09)		25.18 (8.12)	
Living with patient			-0.41		0.49			0.56		-1.48
Yes	73	21.66 (4.6)		16.86 (4.92)		74	69.15 (30.73)		27.73 (8.8)	
No	154	21.38 (4.76)		17.22 (5.46)		155	71.63 (32.6)		25.97 (7.41)	
CarerQoL^a^										
1) Fulfillment care tasks			0.05***		0.1***			0.09***		0.04**
No	11	22.82 (5.27)		19.00 (4.69)		11	84.82 (38.63)		22.73 (12.03)	
Some	103	22.55 (4.53)		18.91 (5.67)		104	80.51 (31.99)		25.48 (7.28)	
A lot of	110	20.47 (4.53)		15.31 (4.25)		111	61.51 (27.79)		28.02 (7.84)	
2) Relational problems			0.1***		0.35***			0.21***		0.01
A lot of	5	27.40 (2.51)		27.40 (7.89)		5	132.8 (35.46)		27.4 (5.77)	
Some	98	22.74 (4.41)		19.93 (4.95)		99	83.03 (28.41)		25.33 (7.59)	
No	124	20.27 (4.55)		14.44 (3.49)		125	58.7 (27.87)		27.41 (8.16)	
3) Mental health problems			0.22***		0.09***			0.16***		0.03**
A lot of	9	27.78 (6.18)		21.62 (5.58)		9	112.44 (32.77)		30.78 (6.74)	
Some	100	23.22 (4.03)		18.34 (5.11)		102	79.98 (29.48)		27.47 (7.17)	
No	119	19.53 (4.09)		15.73 (4.99)		119	59.54 (28.89)		25.39 (8.4)	
4) Care tasks problems			0.22***		0.17***			0.3***		0.01
A lot of	11	27.27 (3.61)		25.40 (6.57)		11	118.18 (34.5)		27.09 (6.38)	
Some	71	23.80 (4.37)		18.69 (4.72)		72	88.67 (26.91)		27.42 (7.81)	
No	145	19.91 (4.10)		15.78 (4.70)		146	58.38 (26.27)		25.98 (8.04)	
5) Financial problems			0.16***		0.08***			0.24***		0.03**
A lot of	5	30.60 (3.36)		26.33 (2.89)		5	125 (9.43)		31.8 (7.95)	
Some	45	24.00 (4.48)		19.20 (5.98)		45	96.16 (31.74)		28.89 (6.5)	
No	178	20.58 (4.27)		16.39 (4.83)		180	62.79 (27.39)		25.78 (8.08)	
6) Support			0.07***		0.04**			0.08***		< 0,01
No	68	22.81 (4.99)		18.15 (5.55)		68	80.1 (33.05)		26.07 (7.89)	
Some	112	21.56 (4.38)		17.34 (5.37)		113	72.25 (30.14)		26.65 (6.77)	
A lot of	46	19.20 (4.28)		15.06 (4.07)		47	53.26 (28.08)		26.79 (10.3)	
7) Physical health problems			0.21***		0.09***			0.18***		0.01
A lot of	19	26.32 (4.74)		20.56 (5.28)		19	101.16 (27.24)		27.68 (7.79)	
Some	97	22.80 (4.22)		18.17 (5.32)		99	78.95 (30.58)		27.47 (6.97)	
No	112	19.50 (4.06)		15.60 (4.77)		112	58.19 (28.03)		25.48 (8.59)	
Carer QoL VAS	228		-0.51***		-0.31***	230		-0.44***		-0.01
CWS-B Support sum^b^	220		-0.21**		-0.21**	222		-0.26***		0.08

*P ≤ 0.05; **P ≤ 0.01; ***P ≤ 0.001. SD: standard deviation. r: correlation coefficients. T: T-statistics (for variables with two categories). η²: one-way ANOVA effect size (for variables with three categories). EOI: Emotional overinvolvement, CC: Critical comments. ^a^The Care Related Quality of Life questionnaire (Carer QoL). Carer QoL-VAS (Visual Analogue Scale), and each question: (1) Fulfilment with carrying out my care tasks, (2) Relational problems with the care receiver, (3) Problems with my own mental health (e.g.,* stress*,* fear*,* gloominess*,* depression*,* concern about the future*), (4) Problems combining my care tasks with my daily activities, (5) Financial problems because of my care tasks, (6) Support with carrying out my care tasks, when I need it, (e.g. from family, friends, neighbors, acquaintances), (7) Problems with my own physical health (e.g.,* more often sick*,* tiredness*,* physical stress*). ^b^Carer Well-being and Support questionnaire version 2, part B - Support scale (CWS-B)

### Patient factors

As displayed in Table 3, negative caregiving appraisals had significantly higher scores for male patients than for female patients. Duration since first psychosis diagnosis showed a significant negative correlation with EOI, and both positive and negative caregiving appraisals. There was a significant positive correlation between patients’ difficulties with mental health and functioning and EOI, CC, and negative caregiving appraisals.

### Relative factors

As displayed in Table 4, females compared to males had significantly higher ratings on EOI, CC, and the negative and positive scales of caregiving appraisals. Relatives with secondary school or less education had significantly higher scores on EOI, CC, and negative caregiving appraisals compared to those with higher education. Household income showed a significant negative correlation with EOI, and both negative and positive caregiving appraisals.

In all the categorical variables of QoL (CarerQoL-7D), when relatives reported *no fulfilment/a lot of problems* compared to those who reported *a lot of fulfilments/no problems*, significantly higher scores were seen for EOI, CC and negative caregiving appraisals. This was also validated with the results from the visual analogue scale of QoL (CarerQoL-VAS). Support from healthcare professionals (CWS) showed a significant negative correlation with EOI, CC and negative caregiving appraisals.

### Hierarchical regression analyses

Table 5 presents the results of the hierarchical regression analyses.

#### Emotional overinvolvement (EOI)

For the EOI outcome, the total model explained 32.7% of the variance. Duration since first psychosis diagnosis (Block 1) was a significant negative predictor of EOI, and patients’ difficulties with mental health and functioning (Block 4) were significant positive predictors. Introducing relatives’ mental and physical health problems in Block 5 made a significant contribution to the model and explained an additional 17.3% of the variance in EOI beyond Blocks 1–4. Adding support from family, friends, neighbours, or acquaintances (Block 6), and support from healthcare professionals (Block 7), did not significantly increase the explained variance (0.7% and 0.4%, respectively).

#### Critical comments (CC)

All seven blocks together explained 12.3% of the variance in CC. Support from healthcare professionals (Block 7) was the only significant predictor in the final model and significantly predicted lower levels of CC. Block 7 made a significant contribution to the model and explained an additional 2.2% of the variance in CC beyond that accounted for by the previous blocks.

#### Negative caregiving appraisals

For negative caregiving appraisals, the total model explained 31.2% of the variance. Duration since first psychosis diagnosis (Block 1) and patients’ functioning (Block 3) were both significant negative predictors with negative caregiving appraisals, whereas patients’ difficulties with mental health and functioning (Block 4) were significant positive predictors with negative caregiving appraisals. Blocks 6 and 7 - support from family and friends, and support from healthcare professionals - each explained a significant additional proportion of variance in negative caregiving appraisals, accounting for a further 2.5% and 1.9% of the variance, respectively, beyond that explained by the previous blocks.

#### Positive caregiving appraisals

Duration since first psychosis diagnosis (Block 1) and household income (Block 2) were significant negative predictors of positive caregiving appraisals. Together, the first two blocks explained almost 10% of the variance in positive caregiving appraisals. Variables entered in Blocks 3–7 did not make a significant contribution to the model and did not meaningfully increase the explained variance.

**Table 5:** Hierarchical regression

	Family Questionnaire (FQ)								Experience of caregiving (ECI)							
	Emotional overinvolvement (EOI)				Critical comments (CC)				Negative scale				Positive scale			
	β	SE	*P*	$\:{\Delta\:}$ *R*_*adj*_^2^	β	SE	*P*	$\:{\Delta\:}$ *R*_*adj*_^2^	β	SE	*P*	$\:{\Delta\:}$ *R*_*adj*_^2^	β	SE	*P*	$\:{\Delta\:}$ *R*_*adj*_^2^
**Block/stage 1**																
Intercept	31,46	2,77	0,000	0.018	24,62	3,80	0,000	-0,008	137,07	18,97	0,000	0,022	29,57	5,35	0,000	0,027
Patient gender (reference=female)	0,81	0,58	0,165		0,09	0,75	0,905		7,01	3,98	0,080		1,89	1,12	0,093	
Years since diagnosed	-0,07	0,03	**0**,**035**		0,03	0,04	0,533		-0,48	0,21	**0**,**027**		-0,13	0,06	**0**,**029**	
**Block/stage 2**																
Relative gender (reference=female)	-1,01	0,61	0,100	0.085	0,72	0,78	0,359	-0,007	0,20	4,17	0,962	0,061	-2,32	1,18	*0*,*051*	0,098
Relative age	-0,02	0,02	0,501		-0,03	0,03	0,358		-0,07	0,16	0,665		-0,02	0,04	0,594	
Income household	-0,13	0,12	0,297		0,23	0,16	0,145		-0,49	0,83	0,556		-0,81	0,24	**0**,**001**	
University education (reference = no)	0,06	0,62	0,928		-0,81	0,81	0,317		-1,24	4,26	0,772		-0,34	1,20	0,777	
**Block/stage 3**																
GAF-F^a^	-0,04	0,02	0,084	0.109	-0,04	0,03	0,195	0,007	-0,50	0,17	**0**,**003**	0,120	0,03	0,05	0,592	0,095
**Block/stage 4**																
Basis-24^b^	1,01	0,40	**0**,**013**	0.143	0,75	0,53	0,164	0,019	7,39	2,76	**0**,**008**	0,157	0,77	0,78	0,320	0,094
**Block/stage 5**																
Some problems mental health (reference=many problems)^c^	-1,77	1,78	0,320	0.316	-1,94	2,44	0,427	0,082	-8,38	12,20	0,493	0,268	-0,53	3,44	0,878	0,079
No problems mental health (reference=many problems)^c^	-4,11	1,85	**0**,**028**		-3,64	2,53	0,152		-19,43	12,71	0,128		-1,45	3,58	0,686	
Some problems physical health (reference=many problems)^d^	-1,53	1,21	0,206		0,16	1,56	0,918		-7,32	8,27	0,377		0,37	2,33	0,873	
No problems physical health (reference=many problems)^d^	-3,05	1,26	**0**,**017**		-1,14	1,63	0,486		-16,88	8,66	0,053		0,48	2,44	0,844	
**Block/stage 6**																
Some support e.g. family (reference = no support)^e^	-0,80	0,64	0,217	0.323	-0,50	0,84	0,548	0,101	-3,13	4,41	0,479	0,293	0,18	1,24	0,887	0,081
A lot of support e.g. family (reference = no support)^e^	-1,36	0,84	0,107		-2,09	1,07	*0*,*053*		-13,84	5,71	**0**,**016**		1,94	1,61	0,230	
**Block/stage 7**																
CWS-B, Support^f^	-0,03	0,02	0,136	0.327	-0,07	0,03	**0**,**019**	0,123	-0,39	0,16	**0**,**015**	0,312	0,07	0,04	0,104	0,09

Bold numbers: p-value ≤ 0.05. β: Beta coefficient. SE: Effect size. ^a^The Global assessment of functioning scale (GAF), split versions for symptoms (GAF-S) and functioning (GAF-F).^b^The Behavior and Symptom Identification scale (Basis-24). Overall mean score. ^c^The Care Related Quality of Life questionnaire (CarerQoL): Problems with my own mental health (e.g.,* stress*,* fear*,* gloominess*,* depression*,* concern about the future*), ^d^CarerQoL: Problems with my own physical health (e.g.,* more often sick*,* tiredness*,* physical stress*). ^e^ CarerQoL: Support with carrying out my care tasks, when I need it, (e.g. from family, friends, neighbours, acquaintances) ^f^Carer Well-being and Support questionnaire version 2, part B - Support scale (CWS-B)

## Discussion

There is a substantial body of literature highlighting the importance of family members as caregivers and their ability to influence outcomes in patients with psychosis [85]. This study presents novel data that both challenge and support prior research regarding family climate. This study highlights that relatives of patients with the shortest duration since first psychosis diagnosis experience the greatest emotional strain, and that perceived professional support can play a key role in reducing criticism and burden. Negative caregiving appraisals and EOI were influenced primarily by the same factors (duration since first psychosis diagnosis, patients’ mental health and functioning, and relatives’ health problems), suggesting that they may arise from similar reactions to stress. Furthermore, EOI and CC were associated with entirely different factors, which have been scarcely described in previous research. Our results are consistent with previous findings regarding the relationship between relatives’ health and EOI. Additionally, our findings emphasise the importance of support-related factors that underpin the ‘stress-appraisal-coping’ model [49, 50].

The level of EOI and CC in this study is low compared to previous studies that have revealed that over half of key relationships between patients and relatives exhibit high levels of EE across different cultural contexts [86]. This may be due to the fact, that in our study only one-third of the relatives lived with the patient, patients were generally years from a first psychosis diagnosis, and patients were recruited from outpatient treatment. This might indicate a relatively stable phase of the illness. Recruiting patients and relatives in pairs likely biased the sample towards more stable relationships; over half of relatives reported no relationship problems with the patient (Table 4), and patients with poor relationships may have been unwilling to participate. This could have affected the proportion of relatives exhibiting high levels of EE.

At inclusion, 17% of the patient/relative pairs had received some level of FPE and 58% of the relatives had received at least one family involvement or support service [87]. Previously received FIs may have increased relatives’ perceived support, improved family communication [88] and reduced EE levels in the sample [18–21].

Western countries emphasise personal autonomy and independence, which can influence relatives’ emotional reactions, caregiver support and expectations of healthcare services when a family member develops a psychotic disorder [89]. High EOI in a relative is often viewed negatively, whereas in more collectivist cultures the absence of EOI may be interpreted as deficient informal care [90, 91]. In Norway, healthcare, practical assistance and accommodation are provided under statutory patients’ rights and largely funded by the welfare system; income is ensured in cases of disability, unemployment or retirement. Although eligibility is strict, included patients may have received healthcare services, supported accommodation, and 53% received financial assistance from the Norwegian Labour and Welfare Administration (Table 1), which may have significantly alleviated the relatives’ burden and EE.

### Patient factors

In line with previous research [92–94], longer duration since the first psychosis diagnosis was associated with a lower level of negative caregiving appraisals. This may be explained by the fact that relatives over time develop more effective coping strategies [95] and as patients grow older and transition to independent living, the patients’ reliance on family support may decrease. Furthermore, longer duration since the first psychosis diagnosis was also associated with a lower level of positive caregiving appraisals. This result aligns with Harvey et al. [96]. They attributed their findings to the intercorrelation between the two appraisal scales of the ECI, as also highlighted by several other studies [97–99]. Research indicates that both negative and positive caregiving appraisals can coexist simultaneously [33, 47], and appear to be strongest early in the course of a psychotic disorder.

McFarlane [68] describes a reciprocal interaction between patient and family factors. Stress-reactive responses to symptoms, perceptions, and behaviours can profoundly impact both the patient and the disorder’s progression over time [68]. As reported in several other studies [44, 96, 98, 100, 101], this study showed that patients’ difficulties with mental health and functioning was associated with higher levels of EOI and negative caregiving appraisals (burden). Levels of EOI may fluctuate over time and typically increase during periods when the patient is experiencing greater difficulties [34, 102–104]. Elevated levels of EOI may represent an emotional response to the concern, stress and anxiety experienced by relatives when their loved ones first encounter psychosis, during periods of exacerbation of the illness, or when the healthcare system fails to meet the patients’ needs [101, 102, 105, 106]. This is an important issue, as many studies have shown that patients living with relatives reporting high levels of EE have an increased risk of relapse [54, 58, 104, 107, 108].

The present study indicates that patient-related factors, including duration since the first psychosis diagnosis, strongly affect relatives’ emotional involvement and burden. This underscores the importance of identifying and supporting families at the onset of psychosis.

### Relative factors

In accordance with a study by Lopez et al. [109], our study found that lower household income was associated with higher positive caregiving appraisals. Lopez and colleagues [97] suggested that balancing caregiving with outside employment can be difficult, potentially increasing stress and reducing satisfaction. In our study, 38% of the relatives were unemployed or retired, suggesting that those with lower incomes may have more time to fulfil their caregiving responsibilities. Norway’s welfare system provides an income safety net for patients and relatives, which may have reduced financial pressure and led to more positive caregiving appraisals among relatives working fewer hours or not at all. In contrast, Koutra et al. [98] argued that non-working relatives with limited financial means are more vulnerable to distress, as restricted access to material and social resources may hinder their ability to meet caregiving demands.

The relatives’ emotional strain from patients’ mental health issues, as previously outlined in the reciprocal interaction [68], can adversely affect their own health [30, 33]. In line with previous research [40, 110, 111], we found that more health problems among relatives were associated with a higher level of negative caregiving appraisals (burden) and EOI. Relatives with high level of EOI tend to attribute the challenges encountered by the patient to external factors beyond the patient’s control [112], and may have a stress-response syndrome [113, 114]. Those heavily invested in their loved ones’ lives take on significant responsibility for supporting them, which can lead to feelings of dependency and substantial losses [87, 103, 115]. This burden can leave them with little energy for self-care activities, including socialising and exercising [94]. Given that the costs often manifest as increased burden, distress, and financial strain, EOI may be more detrimental to relatives than to the patients themselves [110, 116]. Breitborde et al. [111, 117] proposed that EOI could indicate poor health and predict future health deterioration.

This study suggests that relatives’ own health, and resources influence their emotional reactions and caregiving appraisals. Systematic assessment of relatives’ well-being should therefore be an integral part of psychosis services.

### Support

McFarlane [68] pointed out that it is unrealistic to expect families to understand psychotic disorders and to know what to do, independent of professionals’ information, guidance, and emotional support. Our results corroborate this understanding, as perceived support from healthcare professionals was associated with lower CC and negative caregiving appraisals. Weisman et al. [118, 119] suggested that the criticism and anger from relatives with high EE may stem from the belief that patients can and should better control their symptoms, as hypothesised by Hooley [120]. Attributional difficulties among relatives may arise from insufficient information and support, potentially resulting in emotional reactions such as criticism [53, 118, 121, 122].

Relatives of individuals with psychotic disorders are at risk of losing social connections and becoming isolated due to the strain and responsibilities of caregiving over time [123]. Our findings corroborate Szmukler’s ‘stress-appraisal-coping’ model, which posits that social support and service inputs can mitigate negative caregiving appraisals [50, 52]. Both informal and formal support have been found to positively influence caregiving appraisals [48]. Social support is thought to buffer against stress by altering perceptions of the caregiver’s competence and resources, thereby enhancing coping skills [124–126].

In our previous qualitative explorations with patients and healthcare professionals, we described how collaboration and support through FIs can strengthen the family as a resource for the patient. These interventions also showed enhance coping mechanisms of family members [127, 128].

### Clinical implications

Building on existing knowledge and the findings from this study, we identified two key clinical priorities.

First, clinicians need to recognise the relatives’ risk of elevated levels of EOI and negative caregiving appraisals early in the course of psychotic disorders, both of which are common expressions of burden that can have a detrimental impact on the relatives’ health [33]. Practical and emotional support should be tailored to the needs of the patient and their relatives [129]. Rehabilitation interventions aimed at improving the patient’s level of functioning [130], can yield significant benefits for relatives as well [96]. Informal support can influence negative caregiving appraisals, highlighting the role of clinicians in encouraging relatives to maintain their social networks and connect with relevant user organisations and peer support groups [40].

It is important to be aware of the coexistence of both positive and negative caregiving appraisals, as there may be a tendency to view them as exclusively one or the other [116]. Caregiving appraisals seem to be highest in the early stages of psychotic disorders; therefore, clinicians should actively support relatives during this time. Families often express concern, perceiving their loved ones as more vulnerable and at greater risk of relapse in these initial phases [94].

Second, the relationship of relatives’ perceived professional support to reduced criticism further supports research on the power of psychoeducation for preventing or alleviating criticism directed at the patient due to symptoms or illness-related behaviours [18, 131, 132]. Research indicates that more than two FPE sessions can reduce relapse rates for individuals with schizophrenia [11]. Early intervention may help relatives understand and manage their own reactions and needs, thereby enhancing their ability to support and be a resource for their loved ones over time. Clinicians play a pivotal role in providing formal support, including psychoeducation, practical, and emotional support. Early intervention in psychosis services, now established in many countries, offers phase-specific programmes that encourage help-seeking and engagement, provide psychosocial interventions to strengthen family support and to improve insight [133, 134]. EI services have shown better clinical and functional results than treatment as usual [135] also in the long-term, for example reduced risk of suicide at 10-year follow-up [136].

Our findings underscore the necessity of overcoming implementation barriers to routinely offer family involvement and support to patients and their relatives soon after an initial psychosis diagnosis [87, 137, 138]. Healthcare professionals must have access to training and guidance in FIs, including both BFIS and FPE [137, 139]. A proactive, family-oriented approach in the treatment of psychosis is warranted.

### Methodological considerations

The main strengths of this study are the large sample size of patient and relative pairs, the utilisation of reliable and valid instruments, and a comprehensive dataset. It is also unique that we have investigated the factors influencing EOI, CC, negative caregiving appraisals, and positive caregiving appraisals in parallel. A limitation is the cross-sectional design, which precludes evaluation of causal relationships or the direction of effects regarding the association between the included variables. A further limitation is the lack of a psychosis-specific diagnostic interview. The questionnaires were administered to one of the patients’ closest relatives and may not capture the perspectives of other family members, who might offer differing perceptions. Finally, the exclusion of patients who have already received FIs may have skewed the sample to those with reduced access or receptivity to family involvement. Future direction for our analyses could be to investigate the direction of effects between relatives’ EE and caregiving appraisals and various sociodemographic factors and clinical characteristics over time. It will also be possible to analyse subgroups that have received FIs, thereby illuminating how such interventions can influence modifiable variables through longitudinal analyses.

## Conclusion

Psychotic disorders may entail significant burdens and shape the family climate for both patients and their relatives through reciprocal interactions starting early in the course of the illness. Negative caregiving appraisals and EOI may be influenced by the same factors, such as duration since the first psychosis diagnosis and the patient’s mental health and functioning, indicating that these may arise from similar responses to the responsibilities and burdens faced by relatives. EOI and CC should be measured separately as these emotional expressions may be affected by different factors and may necessitate tailored psychoeducation and support. Relatives’ perceived professional support appears to explain a significant amount of variance in CC and negative caregiving appraisals, even after controlling for sociodemographic factors and clinical characteristics. Findings highlight the need for proactive, structured support to relatives early in the course of psychosis to reduce EE-related risk factors and caregiver burden.

## Data Availability

The datasets used and analysed during the current study are available from the corresponding author on reasonable request.

## Abbreviations

EE: Expressed Emotions
QoL: Quality of Life
CMHC: Community Mental Health Centre
IFIP: The Implementation of guidelines on Family Involvement for persons with Psychotic disorders
EOI: Emotional Overinvolvement
CC: Critical Comments
FQ: Family Questionnaire
ECI: Experience of Caregiver Inventory
CarerQoL: Care Related Quality of Life
VAS: Visual Analog Scale
CWS: Caregiver Well-being and Support
BASIS-24: Behaviour and Symptom Identification Scale
GAF: Global Assessment of Functioning scale
PTSD: Post-traumatic Stress Disorder
ANOVA: Analysis of Variance
$\:{R}_{adj}^{2}$: adjusted coefficient of determination
VIF: Variance Inflation Factor
N: Number
SD: Standard Deviation
IQR: Interquartile Range
NOK: Norwegian kroner
r: Pearson’s correlation
β: Beta coefficient for linear regression models
SE: Effect size
P-value: Probability value
FPE: Family Psychoeducation
BFIS: Basic Family Involvement and Support
FIs: Family Interventions
PMFG: Psychoeducational Multi-Family Group
BFT: Behavioural Family Therapy
FFT: Family-Focused Therapy
